# Human iPS Cell-Derived Insulin Producing Cells Form Vascularized Organoids under the Kidney Capsules of Diabetic Mice

**DOI:** 10.1371/journal.pone.0116582

**Published:** 2015-01-28

**Authors:** Sudhanshu P. Raikwar, Eun-Mi Kim, William I. Sivitz, Chantal Allamargot, Daniel R. Thedens, Nicholas Zavazava

**Affiliations:** 1 Department of Internal Medicine, Division of Immunology, University of Iowa, Iowa City, IA, United States of America; 2 Division of Endocrinology & Metabolism, University of Iowa, Iowa City, IA, United States of America; 3 Central Microscopy Research facility, University of Iowa, Iowa City, IA, United States of America; 4 Department of Radiology, University of Iowa, Iowa City, IA, United States of America; 5 Veterans Affairs Medical Center, Iowa City, IA, United States of America; Children’s Hospital Boston/Harvard Medical School, UNITED STATES

## Abstract

Type 1 diabetes (T1D) is caused by autoimmune disease that leads to the destruction of pancreatic β-cells. Transplantation of cadaveric pancreatic organs or pancreatic islets can restore normal physiology. However, there is a chronic shortage of cadaveric organs, limiting the treatment of the majority of patients on the pancreas transplantation waiting list. Here, we hypothesized that human iPS cells can be directly differentiated into insulin producing cells (IPCs) capable of secreting insulin. Using a series of pancreatic growth factors, we successfully generated iPS cells derived IPCs. Furthermore, to investigate the capability of these cells to secrete insulin *in vivo*, the differentiated cells were transplanted under the kidney capsules of diabetic immunodeficient mice. Serum glucose levels gradually declined to either normal or near normal levels over 150 days, suggesting that the IPCs were secreting insulin. In addition, using MRI, a 3D organoid appeared as a white patch on the transplanted kidneys but not on the control kidneys. These organoids showed neo-vascularization and stained positive for insulin and glucagon. All together, these data show that a pancreatic organ can be created *in vivo* providing evidence that iPS cells might be a novel option for the treatment of T1D.

## Introduction

Type 1 diabetes is caused by the destruction of β-cells and can therefore be treated by the replacement of pancreatic β-cells or that of the whole pancreatic organ. The small number of available donors cannot cater for the thousands of patients on the waiting list. To cure diabetes, a variety of immunological application of stem cells is available, for example using bone marrow-derived mesenchymal stem cells or autologous nonmyeloablative hematopoietic stem cell transplantation have been used [1–4]. Recently, Dao’s group reported that human periosteum-derived progenitor cells derived insulin-producing cells ameliorate hyperglycemia in diabetic mouse model [5]. However pluripotent stem cells are more primitive and poorly immunogenic compared to adult stem cell derived progenitor cell. We *hypothesized* that induced pluripotent stem (iPS) cells generated from skin cells can be directed to form IPCs that secrete insulin. Although some progress has been made to generate IPCs using human ES cells, the differentiation process is still very inefficient, expensive and time consuming [6–8]. Moreover, due to current ethical concerns regarding human ES cells, there is a need to develop alternative sources of pluripotent stem cells providing an unlimited source and supply of IPCs. In this regard, the human iPS cells newly generated in our laboratory offer a novel source of pluripotent stem cells that can be made available for generating glucose-responsive IPCs. Here, we report on the generation of human iPS cell-derived IPCs, their characterization and therapeutic potential to correct streptozotocin-induced diabetic and immunodeficient mice.

Currently the success rate of differentiating human ES cells into IPCs is very poor due to a limited understanding of the differentiation process. Consequently the generated cells are usually bihormonal, secreting glucagon and insulin. Recently, the human ES cell-derived IPCs were transplanted into testicular or epididymal fat pads of immunodeficient mice prior to making them diabetic using streptozotocin treatment which selectively destroys the endogenous pancreatic beta cells and corrected hyperglycemia [9,10]. While human ES cells currently remain the gold standard for generating human IPCs, human iPS cells are more appealing because they can be patient tailored[9,11–14]. Recently, several groups reported the mouse iPS cell derived pancreatic β-like cells which can be reverse hyperglycemia in diabetic mouse [15]. Cells derived from iPS cells seem to be less immunogenic when transplanted across MHC barriers [16,17]. Since the IPCs are derived from self, immune rejection should not play a role. However, thus far, the differentiation of human iPS cells to generate IPCs has not been very successful [18]. We therefore hypothesized that pancreatic lineage commitment of human iPS cell-derived definitive endodermal cells enhances their robust differentiation into glucose-responsive and transplantable IPCs. Besides, endodermal cells can be sorted out by their expression of CXCR4, thus eliminating non-differentiated iPS cells that might cause teratomas.

Here, we describe the generation of IPCs using human iPS cells and their potential therapeutic efficacy to correct hyperglycemia in immunodeficient diabetic Rag2^−/−^γc^−/−^ mice. The undifferentiated iPS cells were subjected to differentiation using a multistep protocol to generate IPCs *in vitro*. The successful differentiation of human iPS cells into IPCs was validated by real-time quantitative PCR, immunostaining, transmission electron microscopy and mitochondria stress tests. The therapeutic efficacy of human iPS cell-derived IPCs was investigated in streptozotocin-induced diabetic immunodeficient mice. The real-time fate of the transplanted IPCs was monitored by MRI, which revealed the presence of an organoid on the kidneys that received IPCs. Our *in vitro* differentiation studies, transplantation data glucose tolerance test and reduction in blood glucose levels post IPCs transplantation, suggest that human iPS cell-derived IPCs are glucose responsive and provide long term correction of hyperglycemia without any teratoma formation.

## Results

### Human iPS cells differentiate into insulin producing cells

**Figure 1:**
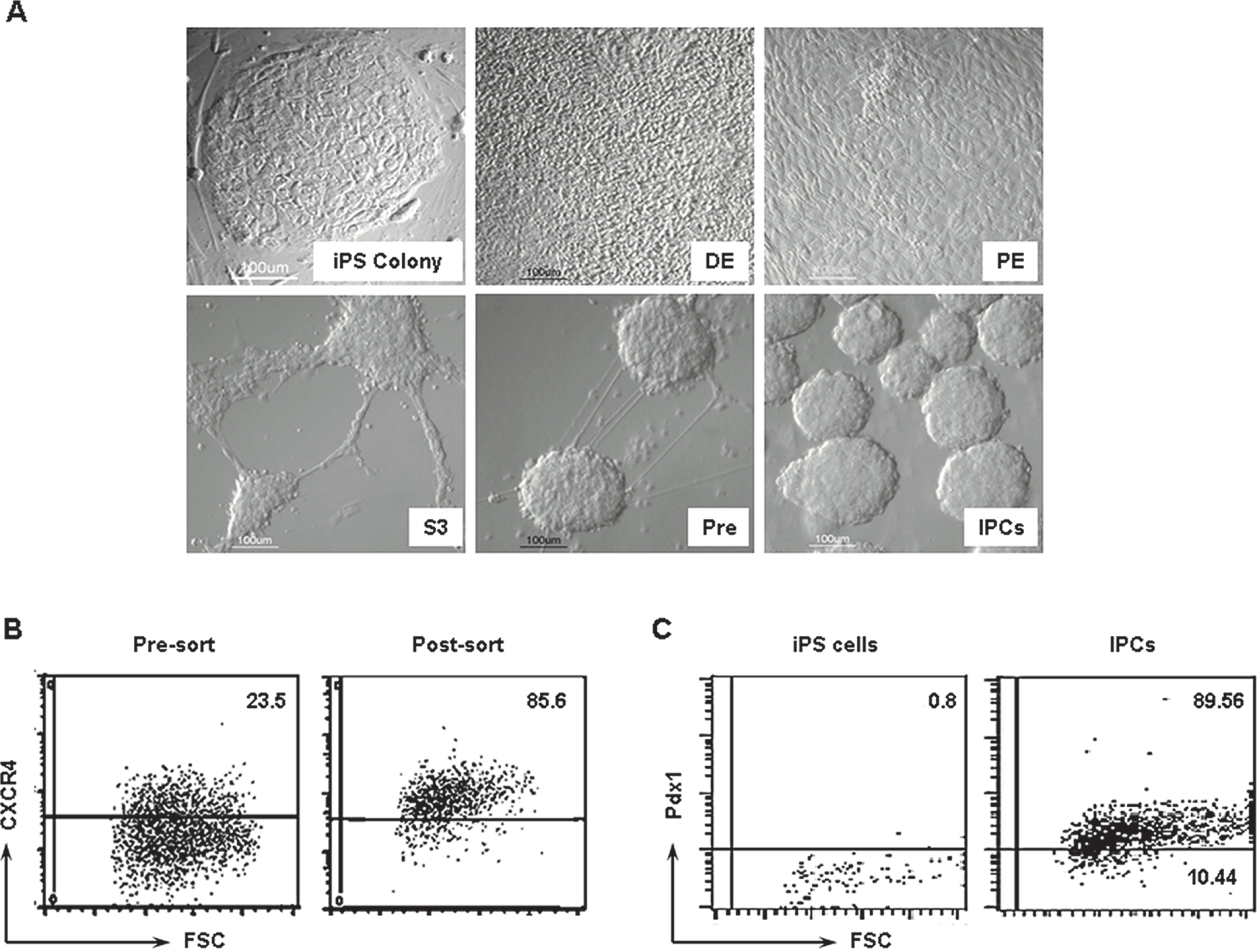
Generation of IPCs. **A)** Stepwise differentiation of human iPS cells leads to the generation of definitive endodermal (DE) cells, pancreatic endodermal (PE) cells, pro-endocrine progenitors (S3), pre-IPCs (Pre) and finally insulin producing cells (IPCs). **B)** At the endodermal stage, the cells were stained for CXCR4 and sorted using immunomagnetic beads. These cells were further used to differentiate IPCs. **C)** Precursor cells were stained for Pdx1 to determine whether they had adopted the pancreatic lineage. Almost all the cells were expressing Pdx1.

Here, we asked the question whether human iPS cells undergo pancreatic lineage commitment to generate IPCs. Human iPS cells were generated as we most recently described [19]. To generate IPCs, a multistep differentiation protocol was used. First, we derived the endoderm by treating human iPS cells with Activin A (Fig. 1A). The endodermal cells express CXCR4, which was exploited to sort the cells out by immunomagnetic bead separation (Fig. 1B). The definitive endodermal cells were allowed to further differentiate and undergo expansion into Pdx1-expressing pancreatic endodermal (DE) cells in the presence of retinoic acid and KGF. Intracellular staining revealed that 89.56% of the pancreatic endodermal (PE) cells were positive for Pdx1 (Fig. 1C). The pancreatic endodermal cells were further cultivated in the presence of HGF and Exendin 4 to generate endocrine progenitors which spontaneously gave rise to three dimensional pancreatic islet like clusters (Fig. 1A). During intermediate stages Pre1 and Pre2, the cells formed projections similar to those of neuronal cells. The morphology of the cells changed during the differentiation procedure. With time the cells transitioned into pancreatic precursors that were polymorphic and flat. Finally, the cells formed cell clusters by day 25. At this stage we termed them IPCs.

To confirm that the cells were pancreatic, we used real time quantitaive PCR to study gene expression during the differentiation process. Our results suggest that during the definitive endoderm formation there is an upregulation of Sox17 and Foxa2 (S1 Fig.). As expected the cells undergoing differentiation transiently expressed high levels of Ngn3.The differentiated cells expressed Pdx1, insulin as well as Glut 2. The human iPS cell-derived IPCs revealed the expression of Foxa2 which has been shown earlier to be important not only for pancreatic beta cell development but also for insulin secretion. The fully differentiated IPCs expressed insulin which was, however, much lower than that detected in fresh human pancreatic islets.

To monitor IPC differentiation *in vitro* in real-time, the undifferentiated human iPS cells were transfected with a vector expressing RIP-Luc. There was no detectable luciferase expression either in definitive endodermal cells or pancreatic endodermal cells. However, differentiation of pancreatic endodermal cells into endocrine progenitors was marked by detectable luciferase expression which increased significantly in the IPCs (S2A Fig.). Progressive differentiation of human iPS cells leads to transcriptional activation of the rat insulin promoter (RIP), which results in significantly enhanced luciferase expression in the IPCs, suggesting secretion of insulin. To further confirm these results, the IPCs were stained with dithizone, which is a sulfur containing compound which binds to metals, including zinc which is found in pancreatic β-cells. The IPCs stained bright red suggesting that they contained zinc (S2B Fig.).

**Figure 2:**
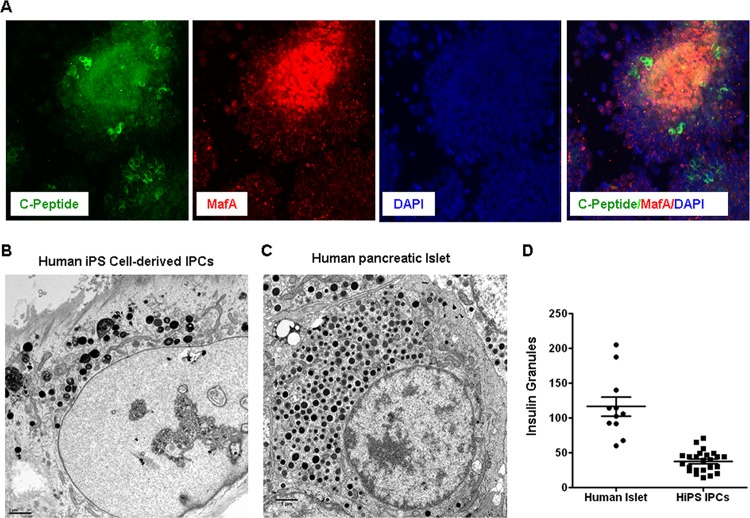
Imaging of IPCs using Transmission Electron Microscopy. **A)** Human iPS cell derived-IPCs were positive for C-peptide as well as Marf A. **B)** Human iPS cell-derived IPCs as well as human pancreatic islets were analyzed by transmission electron microscopy to identify insulin secretory granules. In human iPS cell-derived IPCs a few (∼50–70) insulin granules with and without characteristic halo around them were detected. In contrast, the human pancreatic islets had >100 insulin secretory granules with a characteristic halo around them **(C). D)** The number of granules in both human islets and the IPCs show that IPCs display fewer granules than those counted in the islets.

We further immuno-stained the cells for a number of pancreatic transcription factors including C-peptide, Maf A and glucagon (Fig. 2A, S3 Fig.). We did not stain for insulin as the culture medium contained recombinant insulin. The data show that the cells stained positive for Sox17, Pdx1, C-peptide, Foxa2, Nkx2.2 and glucagon (S3 Fig.). To further characterize the IPCs, transmission electron microscopy of the IPC clusters was performed. IPCs were compared to fresh human pancreatic islets (Fig. 2B-C). IPCs showed electron dense insulin secretory granules characteristic of pancreatic beta cells (Fig. 2D). As expected, IPCs expressed far less granules as compared to β- cells. On average, each IPC contained approximately 20–70 insulin secretory granules compared to 50–200 counted in pancreatic islets. In addition, the insulin secretory granules in the IPCs lacked a characteristic halo, which is typically seen in pancreatic beta cells. This observation potentially suggests that the IPCs are immature at this stage. In addition to the granules, the cells also contained multiple mitochondria indicating an active metabolic phenotype.

### IPC mitochondria consume oxygen after stimulation

**Figure 3:**
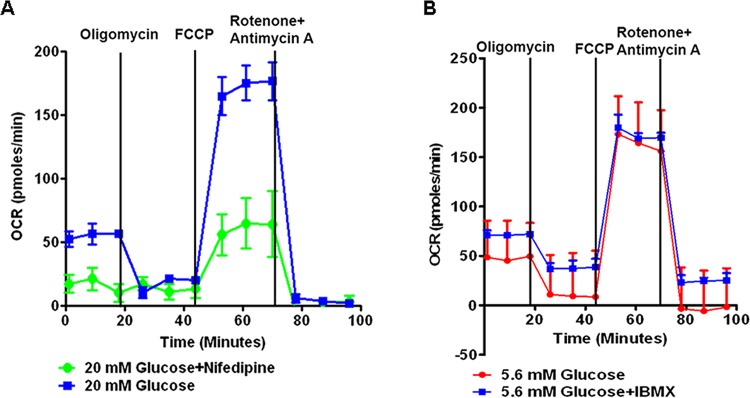
IPCs respond to high glucose levels as detected by the mitochondria stress test. Human iPS cell-derived IPCs were tested for oxygen consumption using the mitochondria stress test. In high glucose **(A)**, oxygen consumption significantly went up and could be blocked by Nifedipine. As expected, in low glucose **(B)** oxygen consumption could not be increased by IBMX (3-isobutyl-1-methylxanthine).

To study the respiration of IPCs, we used the Mitochondria stress test. In high glucose (20mM), the IPCs significantly increased their oxygen consumption (Fig. 3A). As expected, the calcium channel blocker, Nifedipine, successfully inhibited oxygen consumption. Conversely, in low glucose, IPCs consumed oxygen, which could not be elevated by addition of IBMX (Fig. 3B). These results suggest that human iPS cell-derived HPCs respond to glucose levels.

### Human iPS cell-derived IPCs correct hyperglycemia in diabetic mice

**Figure 4:**
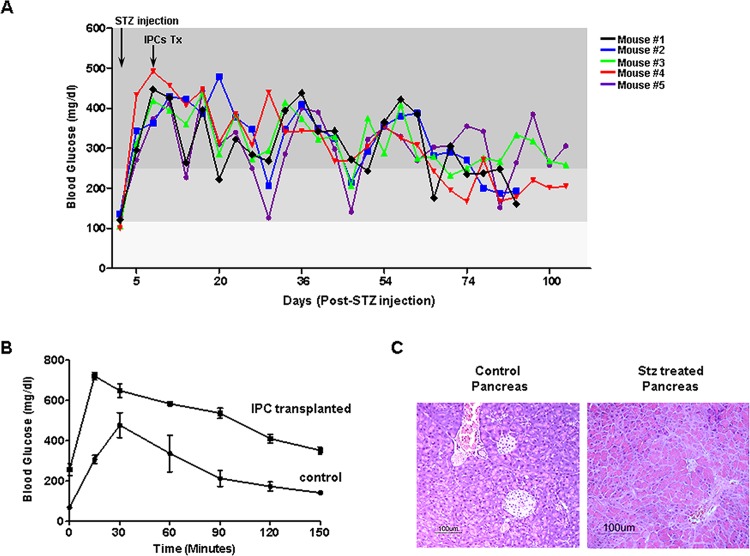
IPCs correct hyperglycemia in diabetic mice. **A)** Eight weeks old Rag2^−/−^γc^−/−^ mice were made diabetic following streptozotocin treatment. The pre-transplant blood levels were >400 mg/dl. Approximately 5×10^6^ human iPS cell-derived IPCs were transplanted under the kidney capsule of each mouse. The blood glucose levels were monitored for >100 days. Blood glucose levels with peak and trough kinetics were observed throughout the duration of the study. The normalization of blood glucose levels was observed in 3 out of 5 mice. The remaining 2 were borderline diabetic. **B)** To study whether the transplanted mice could control high sugar levels, we performed the glucose tolerance test. Mice that had become normoglycemic were subjected to intra-peritoneal glucose tolerance test. The normal healthy control mice displayed a faster blood glucose clearance while the mice transplanted with human iPS cell-derived IPCs had a slightly impaired glucose tolerance. **C)** To rule out that the pancreata of streptozotocin-treated mice did not regenerate, we sacrificed the transplanted mice and histologically examined the pancreas. Streptozotocin-treated mice that had received HPCs did not have any pancreatic islets left.

Next, we asked the question whether the IPCs correct hyperglycemia in diabetic mice. Diabetic Rag2^−/−^γc^−/−^ mice were chosen as recipients of IPCs because they lack a functional immune system. 5×10^6^ IPCs were transplanted under the kidney capsule of STZ induced-diabetic Rag2^−/−^γc^−/−^ mice. The fasting blood glucose levels were monitored regularly over a period of >100 days. The pretransplant blood glucose levels in the STZ induced-diabetic mice were in the range of 400–500 mg/dl. Following IPC transplantation, the fasting blood glucose levels were <200 mg/dl after 100 days in 3 of 6 mice (Fig. 4A). Two mice out of 6 had borderline glucose levels and one mouse died soon after transplantation, and was eliminated from our evaluation. Overall, IPCs effectively regulated glucose levels in all mice. Most of the IPC transplanted mice showed a peak and trough pattern of blood glucose levels indicating that the insulin secretion by the transplanted IPCs may not be fully mature β-cells. These initial results were, however, encouraging. The mice transplanted with IPCs were further subjected to an intra-peritoneal glucose tolerance test at day 100. As compared to the healthy nondiabetic control mice the IPC transplanted mice displayed a poorer blood glucose clearance as compared to control mice (Fig. 4B). The incremental areas under the curve between the control and the IPC transplanted mice were statistically significant, p<0.001, as represented in Fig. 4C. Overall our transplantation data suggest that human iPS cell-derived IPCs are able to regulate hyperglycemia in diabetic mice, however, they are less effective than the pancreas. Further, long-term experiments are required to determine whether the cells continue to secrete insulin over a more extended period than the 150 days studied here and whether their control of hyperglycemia improves with time. As expected, none of the mice developed teratomas.

To rule out that despite streptozotocin treatment the pancreas could have recovered, compromising our IPC results, we histologically examined the pancreas of transplanted mice. Clearly, the pancreas of streptozotocin treated mice was void of any islets as compared to those of control mice (Fig. 4C).

### Real-time noninvasive imaging of transplanted IPCs by MRI

**Figure 5:**
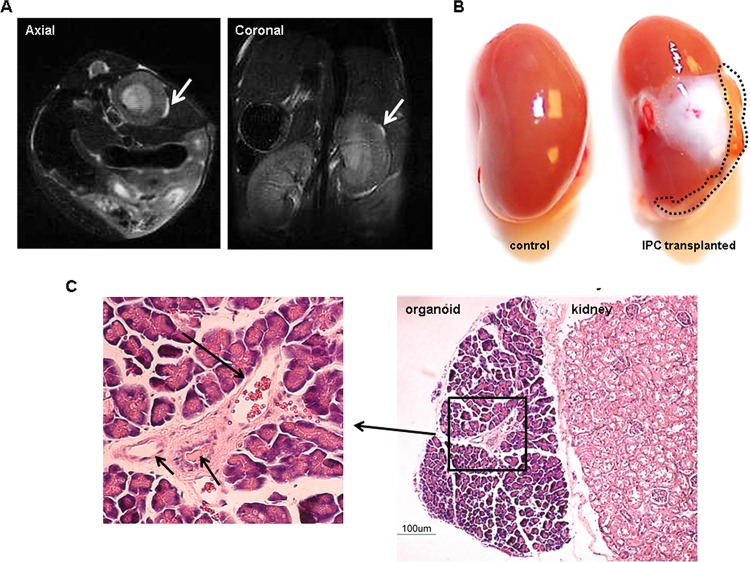
IPCs form organoids *in vivo* under the kidney capsule. **A)** The Rag2^−/−^γc^−/−^ mice transplanted with human iPS cell-derived IPCs were subjected to MRI to monitor the fate of the transplanted IPCs in real-time. MRI revealed that the transplanted IPCs were present as a white mass on the surface of the kidney they were transplanted into. Kidneys were imaged both in the axial and coronal projections. **B)** To further study the tissue on the kidneys, the mice were sacrificed. The transplanted IPCs were observed as a white vascularized organoid with triangular shape. There was no evidence of any teratoma formation or abnormal growth. The IPC transplanted kidney showed signs of tissue adhesion at the site of IPC injection (dotted line). **C)** To examine the organoids further, histological sections were stained by H&E and studied under the microscope. The organoids appeared triangular in shape and separate from the kidneys. More interestingly they appeared to have developed neovascularization (insert, arrow heads).

One of the major caveats in the field of islet transplantation is the lack of noninvasive imaging to monitor the fate and function of the transplanted IPCs. Here, we investigated whether MRI can be used to monitor the long term fate of the transplanted IPCs. MRI imaging was performed 150 days post transplantation. The IPCs transplanted under the kidney capsule could easily be identified by MRI as a dense white mass present on the dorsal surface of the kidney on both the coronal as well as axial projections (Fig. 5A). To exclude the possibility of teratoma formation post-IPC transplantation, the mice were sacrificed and both of the kidneys examined. The transplanted IPC mass appeared as a vascularized triangular area (Fig. 5B). 12 mice were transplanted with IPCs and 10 remained untreated with IPCs but were treated with streptozotocin. These control mice died within 10 days. Besides the IPC transplanted mass, there was an area that showed adhesion to the liver, as a result of the surgical procedure, since we transplanted cells in the right kidney. Gross examination of the kidneys as well as other internal organs revealed the absence of any teratomas. These data confirm that the IPC-derived organoids can non-invasively be monitored by MRI. Further, the data also revealed that the transplantation of human iPS cell-derived IPCs did not lead to teratoma formation thereby highlighting the safety of the human iPS cell-derived IPCs.

To further examine the new pancreatic organoids, the explanted kidneys were stained by H & E. As shown in Fig. 5C, the organoid can be seen next to the kidney but separated by a thin layer of connective tissue. A closer examination further reveals that the organoid had its own blood vessels (insert), suggesting that either the IPCs secreted VEGF or they induced secretion of VEGF by other cells.

**Figure 6:**
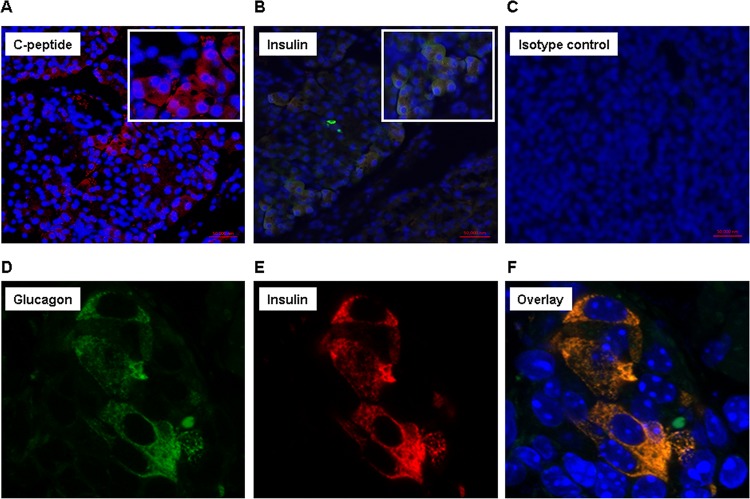
Histology and Immunostaining of the transplanted IPCs. To confirm that the organoids were pancreatic in nature, histological sections were stained for C-peptide **(A)**, insulin **(B, E)**, isotype control **(C)** and glucagon **(D)**. The overlay **(F)** shows that the cells are positive for both hormones suggesting that they are bihormonal.

To confirm that the organoid was pancreatic, histological sections of the explanted kidneys were stained for glucagon and insulin (Fig. 6A). In both cases, the stains were patchy, again stressing the fact that even after 150 days, not all cells were β-cell like. Immature β-cells are bihormonal, secreting both insulin and glucagon. This was highlighted in the overlays that showed evidence of secretion of both hormones. Lastly the sections were stained for C-peptide, which stained in a similar pattern (Fig. 6B). Thus, we show that after 150 days in the kidney capsule, the iPS cell derived cells continued to be pancreatic.

## Discussion

The ability to reprogram somatic cells into pluripotent stem cells is a very appealing approach with the potential to revolutionize future cell-based therapies [20,21]. Here, we show that human iPS cells efficiently generate CXCR4-expressing endodermal cells. By following a step by step approach, we successfully generated pancreatic precursor cells that were Pdx1^+^, a master transcription factor that regulates the development of the pancreas [22]. We and others have tried different approaches for generating IPCs with mixed results. For example, we directed mouse ES cells towards IPCs, but were never able to eliminate partially differentiated cells. Consequently some of the mice that were transplanted with these cells developed teratomas [23]. In some cases the process was barely efficient in generating IPCs. Here, the IPCs formed cell clusters at the end of the differentiation process. These cell aggregates could be counted and transplanted into diabetic mice.

The gene expression of the differentiating cells was followed by quantitative real time PCR. IPCs clearly had elevated insulin, Sox17 and Pdx1. The levels of Glut2 were quite low, suggesting that the IPCs may still not be fully mature and might poorly respond to glucose challenge. In comparison to pancreatic islets, the level of insulin in the IPCs was relatively low. Indeed when we performed transmission electron microscopy of the IPCs and human islets, IPCs contained only a third of the zinc containing granules compared to those found in islets. These data suggested that freshly differentiated IPCs poorly secrete insulin. These IPCs, however, underwent mitochondrial stress testing showing a good response to high glucose levels, which could be blocked by Nifedipine. Thus, while the cells at this stage were not as robust as pancreatic islets, they responded to glucose.

Interestingly, diabetic mice responded well to the transplantation of IPCs. It is worth noting that after IPC transplantation, the glucose levels were going down and rebounding up again. It is not clear whether this reflects the immaturity of our cells and their poor response to glucose or whether some other metabolic control mechanisms are involved. We ruled out the possibility that the peaks could have been caused by eating times when the mice were feeding, because mice were fasted before the glucose levels were measured. Others have noted the same phenomenon with ES cell-derived IPCs [24]. However, after mice became normoglycemic by day 100, glucose levels showed a steady level with no rebounds. We *hypothesize* that the cells had matured and were better able to regulate glucose levels at this stage. The glucose tolerance test showed a gap between control mice and mice that received IPCs. Control mice more efficiently controlled glucose levels. Our histological data on the pancreas of the transplanted mice confirmed that streptozotocin had in fact destroyed the mouse’s own β-cells. Thus, serum glucose levels of the transplanted diabetic mice can be attributed to the IPCs.

We were surprised to discover in Fig. 5C that IPCs had formed 3D vascularized organoids. It is unclear what initiates the vascularization. The most likely scenario is that the cells secrete VEGF and perhaps other growth factors that promote vascularization. The kidney looked intact and nicely separated from the organoid which had acquired a thin capsule. To our knowledge, this is the first pancreatic organoid to be generated from human IPS cells and is able to regulate serum glucose levels. The kidney capsule has been used by many other researchers for the transplantation of human pancreatic islets [8,25] and has proven to be an excellent site for the transplantation of islets to treat diabetes. In our case, IPCs seemed to form an independent organoid with its own blood vessels. To further characterize these new tissues, we need to further determine whether there are neo-ducts in the tissue. Further, DNA array data could provide invaluable data on the gene expression pattern of these new tissues.

The new organoids stained positive for insulin and glucagon. Thus, we have established cells that produce endocrine hormones. Fig. 6F shows that the cells appear to be bihormonal, a characteristic of immature pancreatic cells. We recognize that these stains were patchy in the tissue and that the intensity varied, supporting the idea that some cells were more advanced in their maturation and others were not. These data are a first step towards iPS cell-derived IPCs. The protocol needs to further improve towards generating cells that secrete insulin only. Our data are consistent with published data, which suggest that IPCs appear to mature post-transplantation in the *in vivo* environment [8,26,27]. Others have supplemented the transplantation of IPCs inserting insulin chips in the recipient mice early post transplantation [24]. It is unclear whether exogenous insulin contributes to the maturation of IPCs. Although the maturation of IPCs remains a challenge, we are encouraged by the ability to generate an organoid that is fully vascularized and controls serum glucose levels.

Recently, another protocol was reported which led to the generation of IPCs that corrected hyperglycemia in both mice and rats [24]. Schulz et. al. showed that ES cell-derived IPCs could be produced on a large scale [27]. Both reports show that the IPCs maturate *in vivo* after 4–5 months. This is consistent with our own data. However, it is exciting that we created a 3D organoid that secretes insulin *in vivo* for the first time. Our data encourage further advances in this field which could ultimately lead to a therapeutically applicable protocol. Most recently after submission of this manuscript, two new manuscripts showed that human IPCs generated from iPS cells required a shorter time than previously reported for IPCs to correct hyperglycemia [28,29]. This is exciting and should lead to improved management of type 1 diabetes.

## Materials and Methods

### Differentiation of human iPS cells into IPCs

Undifferentiated human iPS cells at passage 28 were maintained on irradiated primary mouse embryonic feeder cells until they formed individual colonies. The undifferentiated iPS cell colonies were subjected to a multistep differentiation protocol. Initially, the iPS cells were treated with serum free DMEM/F12 supplemented with Activin A (100 ng/ml) and Wnt3a (25 ng/ml) for 24 hours. Subsequently, the cells were treated with DMEM/F12 supplemented with 100 ng/ml Activin A and 0.2% FBS for 4 days to allow their robust differentiation into definitive endodermal (DE) cells. The DE cells were trypsinized to generate single cell suspension and plated them (3×10^4^ cells/cm^2^) onto gelatin coated 6 well plates. The cells were maintained in DMEM/F12 supplemented with retinoic acid (2 μM), keratinocyte growth factor (25 ng/ml), Noggin (50 ng/ml), 0.5% ITS, 2% B27 (contained with recombinant insulin) without vitamin A, 1% non-essential amino acids, 1% glutamax and 0.1 mM β-mercaptoethanol for 6 days to generate pancreatic endoderm. The pancreatic endoderm thus generated was cultured in DMEM supplemented with HGF (20 ng/ml), exendin-4 (50 ng/ml), nicotinamide (10 mM) for 6 days to generate pancreatic endocrine precursors. The pancreatic endocrine precursors were allowed to mature and undergo expansion in DMEM supplemented with 10% FBS and nicotinamide (10 mM) for 8–10 days until further characterization or transplantation.

### Mice

All animal experiments were approved and performed according to International Animal Care and Use Committee (IACUC) guidelines. The University of Iowa animal vivarium is accredited by the Association for the Assessment and Accreditation of Laboratory Animal Care (AAALAC). Eight week-old Rag2^−/−^γc^−/−^ male mice (Jackson Laboratory, Bar Harbor, ME, USA) were used for the transplantation experiments. Diabetes was induced by five consecutive intraperitoneal streptozotocin (STZ) (EMD Millipore Corporation, Billerica, MA< USA) injections (40 mg/Kg body weight). STZ was reconstituted in ice cold fresh sodium citrate buffer (pH 4.5) immediately prior to injection. The fasting blood glucose levels were regularly monitored using a HemoCue glucose 201 analyzer (HemoCue AB, Ängelholm, Sweden). Mice with blood glucose levels >350 mg/dl for two consecutive readings that were five days apart were considered diabetic. Diabetic Rag2^−/−^γc^−/−^ mice do not survive beyond 15–20 days due to severe hyperglycemia. Approximately 5 × 10^6^ human iPS cell-derived IPCs were transplanted under the kidney capsule of the diabetic mice as described earlier [23]. The IPC transplanted mice were kept under observation for 150 days and their blood glucose profiles were monitored on weekly intervals.

### Bioluminescence imaging

The undifferentiated human iPS cells were transfected with pGL4/RIP-Luc vector and their *in vitro* differentiation into IPCs at various stages was monitored by real-time bioluminescence imaging as described earlier [30]. The relative Luciferase expression at various stages was calculated and the results were analyzed by GraphPad prism 5.

### Transmission electron microscopy

To confirm whether the human iPS cell-derived IPCs are indeed producing insulin, the IPCs were subjected to transmission electron microscopy. To facilitate the identification of IPCs, the cells were subjected to real-time bioluminescence imaging first and the cells displaying the robust bioluminescence signal were considered insulin producing and were processed for transmission electron microscopy. As a positive control we used the human pancreatic islets made available through the City of Hope Integrated Islet Distribution Program. Briefly, the IPCs or the human pancreatic islets were fixed in 2.5% wt/vol glutaraldehyde for 24 hours. The fixed IPCs and the islets were treated with 100 mM cacodylate buffer (pH 7.4) containing 3% wt/vol formaldehyde, 1.5% wt/vol glutaraldehyde for 15 minutes. The fixed IPCs or the pancreatic islets were subjected to osmification in 1% Osmium tetroxide and then stained with Uranyl acetate after washing step. The cells were subsequently dehydrated through a series of graded ethanol solutions and embedded in Epon. The embedded cells were cut into 30μM thin sections using glass knife on a Leica Ultramicrotome and analyzed on JEOL JEM-1230 Transmission electron microscope. The images were acquired using Gatan Ultrascan CCD camera and the images were analyzed by using Image J program.

### Mitochondria stress test

Oxygen consumption rate (OCR) was measured by using an intact-cell respirometer designed for adherent cells (Seahorse Bioscience, North Billerica, MA). Human iPS cell-derived IPCs were grown in special 24-well plates designed for respirometer analyses. OCR was determined in assay medium consisting of medium M199 lacking sodium bicarbonate for 60 min. Before analysis, IPCs within individual wells were exposed to either, low glucose (2.8 mM), high glucose (20 mM), high glucose (20 mM) + Nifedipine. During respirometry, wells were sequentially injected at the times indicated in the figures with oligomycin (2 μM) to block ATP synthase to assess respiration required for ATP turnover (OCR_ATP_), carbonyl cyanide *p*-[trifluoromethoxy]-phenyl-hydrazone (FCCP; 2 μM), a proton ionophore, to induce chemical uncoupling and induce maximal respiration, or antimycin A (0.5 μM) plus rotenone (2 μM) to completely inhibit electron transport and measure non-mitochondrial respiration. The FCCP concentration used in these studies was determined by titration with differing amounts of the uncoupler by using the least amount required for maximal uncoupling in cells unexposed to MTQAs. OCR (pmol per minute per microgram of DNA) was determined as the average number recorded during time periods defined as intervals between the above sequential injections. Basal OCR was determined as respiration before injection of any compounds minus non-mitochondrial OCR. OCR_ATP_ was determined as basal OCR minus OCR after oligomycin injection. OCR accountable by the proton leak was calculated as OCR in the presence of oligomycin minus non-mitochondrial OCR. Maximal uncoupled respiration was calculated as OCR after FCCP minus non-mitochondrial OCR. All values for OCR were normalized to DNA content of the individual wells. ECAR was quantified simply as the recorded acidification rate during the respiratory conditions delineated above.

### Immunostaining and confocal microscopy

The human iPS cells grown on chambered glass slides were differentiated into IPCs and were fixed with 2% paraformaldehyde, quenched in PBS containing 30mM glycine and permeabilized with 0.1% Triton X-100 for 30 min at RT. The cells were stained with primary antibodies against Foxa2 (SC-6554, goat polyclonal IgG, Santa Cruz Biotechnology, Santa Cruz, CA), Sox17 (SC-17356, goat polyclonal IgG, Santa Cruz Biotechnology, Santa Cruz, CA), glucagon (SC-13091, rabbit polyclonal IgG, Santa Cruz Biotechnology, Santa Cruz, CA), insulin (SC-7838, goat polyclonal IgG, Santa Cruz Biotechnology, Santa Cruz, CA) respectively. The cells were visualized by the use of either the Alexa Fluor 488 conjugated donkey anti-rabbit (A21206, Molecular Probes, Invitrogen, Carlsbad, CA) or Alexa Fluor 546 conjugated donkey anti-goat (A11056, Molecular Probes, Invitrogen, Carlsbad, CA) secondary antibodies. Multiphoton imaging was performed on Zeiss LSM 710 microscope using 20X objective lens and the images were captured as grayscale pictures and processed using the ZEN 2011 imaging software. Immunohistochemical analysis and hematoxylin-eosin staining of the tissue sections was performed.

### Intra-peritoneal glucose tolerance test (IPGTT)

IPGTT was performed as we recently described [8]. Briefly, the control and IPC transplanted mice were fasted overnight for 16 hours. Next morning, the body weights were calculated, their fasting blood glucose values were monitored and each mouse was injected with a glucose solution intraperitoneally at a dose of 2g/kg body weight. Thereafter, the blood glucose levels were monitored at a regular interval of 30 minutes up to a maximum duration of 180 minutes. The blood glucose values were plotted as a function of time and the incremental area under the curve were calculated.

### Statistical analysis

The experimental data were analyzed using the GraphPad Prism 5 software (GraphPad Software, Inc., San Diego, CA, USA). The data were tested for significance with Student’s *t*-test or one-way ANOVA where applicable. In all cases, *P <0.05 was considered significant.

## Supporting Information

S1 Fig Gene expression at various stages during the differentiation. Real-time quantitative PCR analysis at different differentiation stages revealed stage specific upregulation of genes involved in pancreatic beta cell development. In this diagram S1 represents DE cells, S2 represents PE cells, S3 represents pro-endocrine progenitors and Pre represents pre-IPCs. Human pancreatic islets and mouse βTC3 cells were used as positive controls while the T cell line NIH3T3 was used as negative control.
(TIF)

S2 Fig Characterization of IPCs. **A)** Real-time bioluminescence imaging and Dithizone staining were performed to confirm the generation of IPCs. The undifferentiated human iPS cells were transfected with a RIP-Luc expression vector. The cells at various stages of differentiation were subjected to real-time bioluminescence imaging. The undifferentiated cells as well as DE cells (S1) failed to generate detectable bioluminescence signal. However, very faint bioluminescence signal was detected in the PE cells (S2). The bioluminescence signal intensity progressively increased in the pro-endocrine progenitors (S3) and maximum bioluminescence signal intensity was observed in the human iPS cell-derived IPCs. **B)** The human iPS cell-derived IPCs were stained with dithizone stain. The IPCs strongly stained positive.
(TIF)

S3 Fig Immunostaining of IPCs. The human iPS cells undergoing differentiation were subjected to immunostaining at various stages. The differentiation led to generation of DE cells which were positive for Sox17 and Foxa2 **(A)**. The PE cells were positive for Pdx1 and Nkx2.2 **(B)**. The islet-like clusters were positive for C-peptide as well as glucagon **(C)**.
(TIF)
