# Supplementary figures and images for: Human iPS Cell-Derived Insulin Producing Cells Form Vascularized Organoids under the Kidney Capsules of Diabetic Mice
Source: PLoS One. 2015 Jan 28;10(1):e0116582. doi: 10.1371/journal.pone.0116582 (PMC4309616; doi:10.1371/journal.pone.0116582)

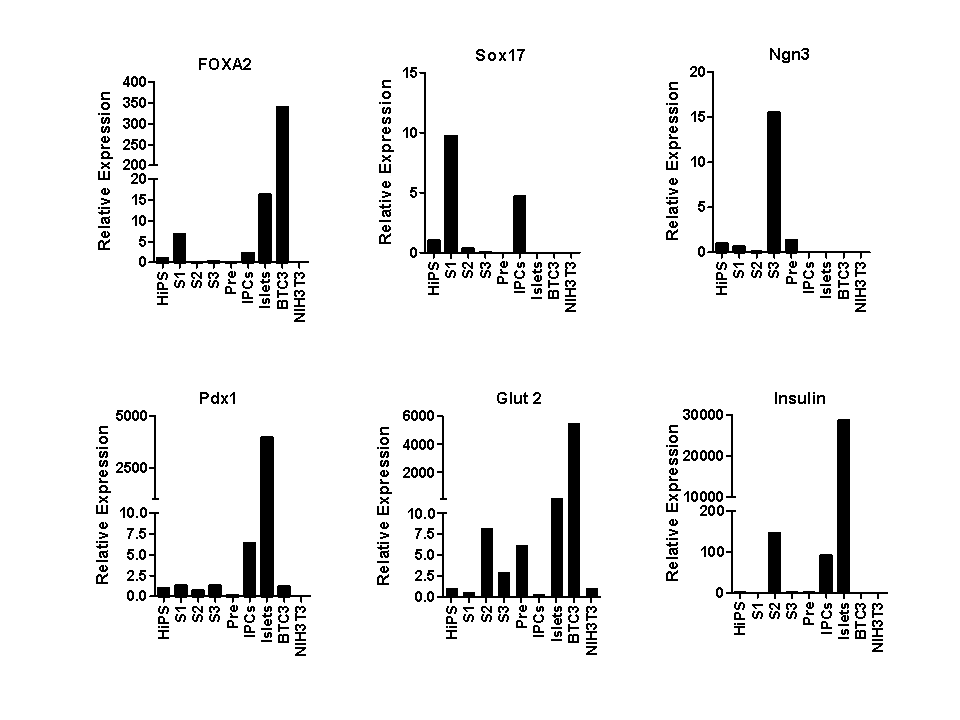

Supplement: S1 Fig — Real-time quantitative PCR analysis at different differentiation stages revealed stage specific upregulation of genes involved in pancreatic beta cell development. In this diagram S1 represents DE cells, S2 represents PE cells, S3 represents pro-endocrine progenitors and Pre represents pre-IPCs. Human pancreatic islets and mouse βTC3 cells were used as positive controls while the T cell line NIH3T3 was used as negative control. (TIF) [file pone.0116582.s001.tif]

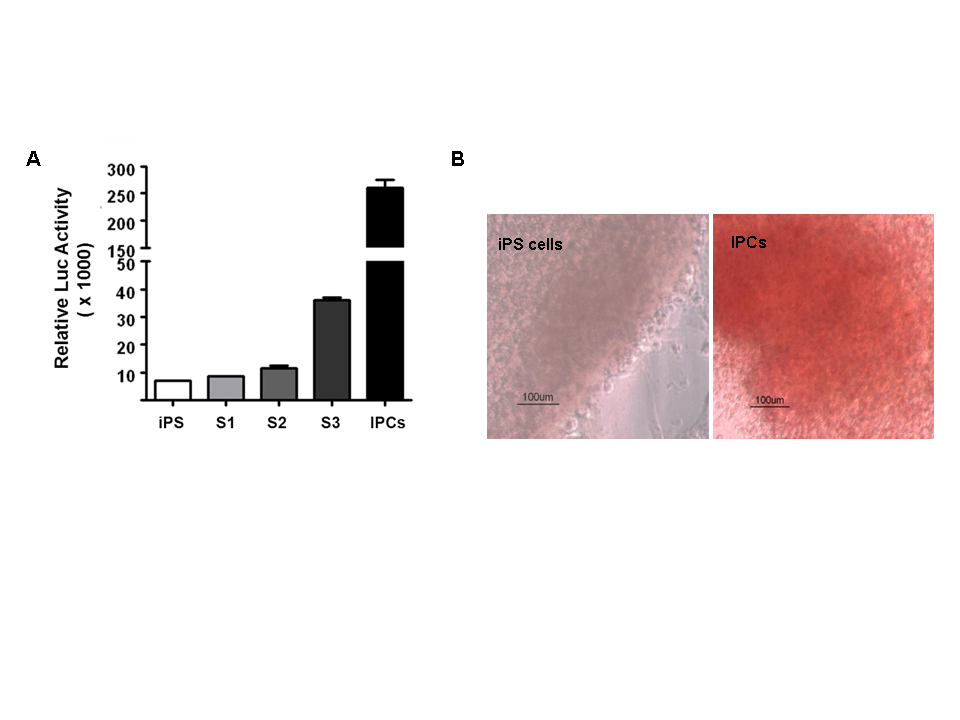

Supplement: S2 Fig — A) Real-time bioluminescence imaging and Dithizone staining were performed to confirm the generation of IPCs. The undifferentiated human iPS cells were transfected with a RIP-Luc expression vector. The cells at various stages of differentiation were subjected to real-time bioluminescence imaging. The undifferentiated cells as well as DE cells (S1) failed to generate detectable bioluminescence signal. However, very faint bioluminescence signal was detected in the PE cells (S2). The bioluminescence signal intensity progressively increased in the pro-endocrine progenitors (S3) and maximum bioluminescence signal intensity was observed in the human iPS cell-derived IPCs. B) The human iPS cell-derived IPCs were stained with dithizone stain. The IPCs strongly stained positive. (TIF) [file pone.0116582.s002.tif]

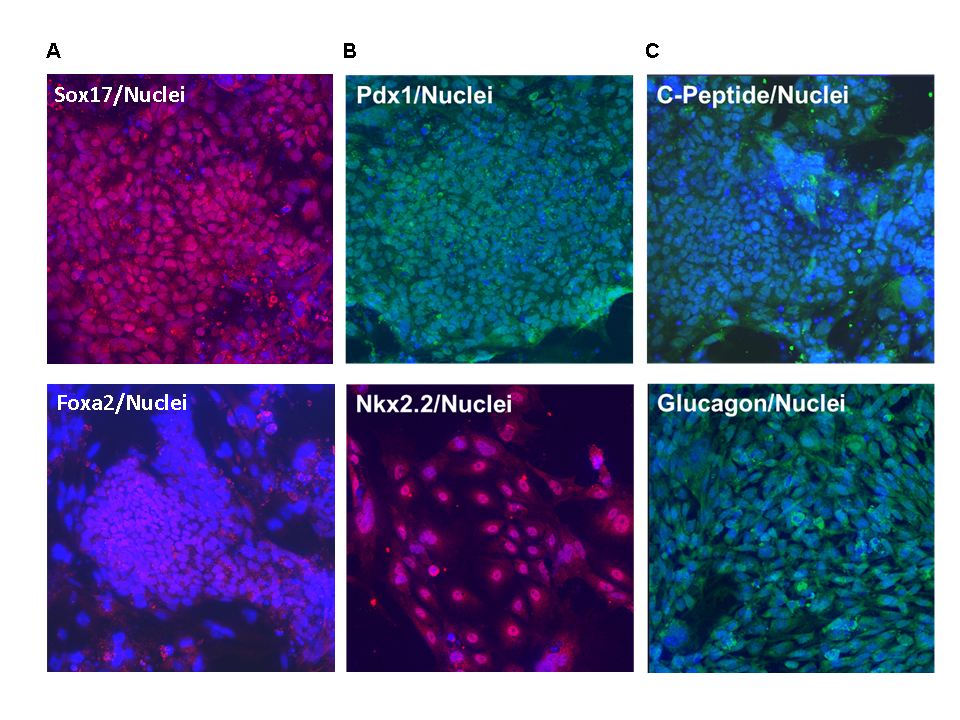

Supplement: S3 Fig — The human iPS cells undergoing differentiation were subjected to immunostaining at various stages. The differentiation led to generation of DE cells which were positive for Sox17 and Foxa2 (A). The PE cells were positive for Pdx1 and Nkx2.2 (B). The islet-like clusters were positive for C-peptide as well as glucagon (C). (TIF) [file pone.0116582.s003.tif]
